# Seasonal Variations in Training Load, Sleep Parameters, and Hormonal Markers in Collegiate Male Rowers During the Off-Season

**DOI:** 10.3390/sports13110407

**Published:** 2025-11-12

**Authors:** Junta Iguchi, Masaki Takimoto, Kenji Kuzuhara, Tatsuya Hojo, Yoshihiko Fujisawa

**Affiliations:** 1 Department of Health and Sports Sciences, Faculty of Health and Medical Sciences, Kyoto University of Advanced Science, Kyoto 621855, Japan; takimoto.masaki@kuas.ac.jp; 2 Department of Athletic Training and Conditioning, School of Health and Sport Sciences, Chukyo University, Toyota 4700393, Aichi, Japan; k-kuzuhara@sass.chukyo-u.ac.jp; 3 Faculty of Health and Sports Science, Doshisha University, Kyoto 6100394, Japan; thojo@mail.doshisha.ac.jp (T.H.); yfujisaw@mail.doshisha.ac.jp (Y.F.)

**Keywords:** training load, sleep parameters, seasonal variation

## Abstract

Background: Adequate sleep is essential for recovery and performance in athletes. Internal and external training loads closely relate to sleep, but few studies have examined their distinct off-season effects. This study investigated the relationships among training load, hormonal markers, and sleep parameters in collegiate male rowers. Methods: Eleven rowers were monitored over 4 months (October 2022–January 2023). Internal load was assessed via session ratings of perceived exertion, external load by accelerometry-based indicators, sleep variables by actigraphy and the Pittsburgh Sleep Quality Index, and hormonal status via salivary cortisol and testosterone. Repeated-measures analysis of variance and correlation and regression analyses were applied. Results: Training load showed significant temporal variation (*p* < 0.01), with October having the highest intensity. Increased loads were associated with poorer sleep outcomes, including reduced total sleep time (*p* < 0.05), higher latency (*p* < 0.05), and decreased efficiency (*p* < 0.05). External load was linked to fragmentation indices (wake after sleep onset, awakenings; *p* < 0.05), whereas internal load correlated with improved efficiency in certain months (*p* < 0.05). Hormonal fluctuations, particularly cortisol and testosterone, also correlated with sleep parameters (*p* < 0.05). Conclusions: Internal and external loads exerted distinct influences on sleep, supporting the hypothesis that both must be monitored. Sleep duration and efficiency emerged as modifiable targets for optimizing recovery and performance in athletes.

## 1. Introduction

Adequate sleep is essential for optimal athletic performance, recovery, and overall health [1]. In particular, post-exercise recovery aims to reduce fatigue and facilitate training and competition performance, and sleep plays a vital role in supporting these processes [2]. Thus, in recent years, the importance of sleep among athletic populations has gained increasing attention. In fact, sleep has been identified as the most favored recovery strategy among athletes across various sports in cross-sectional studies [3].

However, athletes tend to have lower sleep quality and quantity than the general population [1,4]. This phenomenon may be attributed not only to intense training and psychological stress, but also to sport-specific factors such as early morning or evening competition schedules [5]. Sleep disturbance (<7 h) negatively affects not only athletic performance but also various physical and psychological aspects, including an increased risk of injury [6] and mental health issues [1]. Conversely, optimal sleep not only promotes recovery but also contributes to enhanced performance and training adaptations [2].

In recent years, an increasing number of studies have sought to quantify training load from various perspectives, especially by classifying it into internal and external loads. As defined by Soligard et al. [7], external load refers to any measurable external stimulus imposed on the athlete, independent of their internal factors, whereas internal load denotes the individual’s physiological and psychological response to the external stimulus, depending on biological and environmental factors. Similar to sleep, excess training load can be a risk factor for injuries; in contrast, loads managed appropriately can improve performance [8].

Accumulating evidence suggests that training load may influence sleep parameters such as sleep duration, quality, and efficiency [5]. In addition, multiple studies have confirmed that athletes’ physiological and performance parameters exhibit seasonal variations in response to changes in training load across different sports [9,10]. For instance, significant seasonal fluctuations in aerobic and anaerobic capacity, strength, agility, body composition, and flexibility have been observed in elite soccer players [11], and similar seasonal changes in physical fitness and physiological profiles have been reported among Paralympic athletes [12]. Together, these findings underscore that performance-related parameters fluctuate throughout the season in response to training load, competition demands, and recovery patterns. This emphasizes the importance of monitoring seasonal trends in athlete conditioning and well-being. Load monitoring (i.e., recording and collecting training load), has recently attracted attention and is effective for understanding athletes’ conditioning and readiness for competition; it is also considered to help prevent overreaching and injuries [13]. Given the close, mutual relationship between sleep and training load, we believe that they are in a trade-off relationship and that both are modifiable factors. Therefore, accurate tracking and effective management of these factors are paramount to ensure optimal athletic performance.

As described above, some evidence indicates that increased training loads are associated with sleep disturbances [5]; however, few studies have clearly distinguished between the effects of internal and external training load on sleep, and it remains unclear which sleep-related parameters are influenced by each type of load. Additionally, comprehensive studies examining seasonal variations in training load and sleep parameters, particularly over multiple months during the off-season, are limited. As sleep and training load are modifiable factors that can be optimized to enhance recovery and performance, understanding their dynamic relationship is critical. Therefore, this study investigated the relationships between internal and external training loads and sleep-related parameters in collegiate male rowers during the off-season, including hormonal markers. We hypothesized that increased training load worsens sleep quality and quantity; internal and external loads affect sleep differently; and appropriate training load is associated with adequate sleep.

## 2. Materials and Methods

### 2.1. Participants & Study Design

On the basis of parameters derived from a prior study with a comparable design [14], we conducted an a priori power analysis using G*Power^®^ (version 3.1.9.6; Heinrich Heine University, Düsseldorf, Germany). Assuming a large effect size (partial η^2^ = 0.15, equivalent to Cohen’s f = 0.447), a significance level of 0.05, and three repeated measures, the analysis indicated that a minimum of 10 participants would be sufficient to achieve 80% statistical power in a within- participants repeated-measures analysis of variance (ANOVA). Eleven collegiate male rowers (mean ± standard deviation age = 21.2 ± 0.8 years; range: 20.0–22.0) from the same university rowing club, most of whom started rowing at university, were recruited (Table 1). The study employed a longitudinal repeated-measures design, and participants took part for 4 months (16 weeks) from October 2022 to January 2023, including a 1-week winter break in December. Participants were eligible if they were male collegiate rowers aged 18 years or older, engaged in regular training (≥4 sessions per week), and medically cleared for full participation. Athletes were excluded if they had musculoskeletal injuries or medical conditions that restricted training, diagnosed sleep disorders, used sleep or hormonal medications, or failed to comply with study procedures. This research received ethical approval from the Kyoto University of Advanced Science’s institutional review board (approval number: 22M01). Prior to participation, participants were informed of the potential risks and benefits related to this study and provided written informed consent. During the first week of each month (October 2022–January 2023), participants completed a standardized monitoring week. After each session, session RPE and training duration were recorded. A triaxial accelerometer was worn continuously during the week to capture external load and sleep, and participants also completed a daily sleep log and the PSQI at the end of the week. Saliva was collected on Monday afternoon (team day off) for cortisol and testosterone analysis.

### 2.2. Training Load Monitoring (Internal and External Training Load)

All participants were instructed to record their training time and rate the intensity of sessions using the modified Borg Category-Ratio-10 scale (rating of perceived exertion [RPE]), within 30 min after the end of each session during a designated 1-week monitoring period scheduled for the first week of each month [15]. Internal workloads were calculated by multiplying the session RPE by the training time in minutes. Regarding external workload (accelerometry-based external load indicators [ABELIs]), Gómez-Carmona et al. [16] measured external load during matches using multiple 3D accelerometers (RealTrack Systems, Almeria, Spain) and reported a very high correlation (r > 0.803, *p* < 0.01) between different ABELIs when standardized (Z-score transformed) data were used. Accordingly, we obtained ABELIs via a three-axis accelerometer (wGT3X-BT; ActiGraph^®^, Pensacola, FL, USA) worn by participants, which measured the effect of gravitational forces and changes involving movement along the vertical, mediolateral, and anteroposterior axes (x, y, and z, respectively) of a body segment (unit: g) [16]. The three-axis accelerometer data were processed with ActiLife^®^ software (ver. 6SE; ActiGraph^®^, Pensacola, FL, USA; sampling frequency, 30 Hz) [16]. ABELIs were calculated as follows:

$$\Sigma \sqrt{x^{2}+y^{2}+z^{2}}$$

The accelerometer data, collected in the same week as the internal data, were analyzed using Python^®^ 3 (Python Software Foundation, Wilmington, DE, USA) on the basis of the recorded training time.

### 2.3. Sleep-Related Variables

All sleep-related variables, except the Pittsburgh Sleep Quality Index (PSQI) and sleep log, were recorded using the wGT3X-BT three-axis accelerometer (ActiGraph^®^, Pensacola, FL, USA), which participants were instructed to wear throughout the day. The accelerometer provided the total minutes in bed (TMB; time between going to bed [getting in bed] and waking [getting out of bed]), total sleep time (TST [min]; time spent asleep, measured from sleep onset to sleep end), wake after sleep onset (WASO [min]; duration of wakefulness occurring during sleep), number of awakenings during sleep, sleep latency (min; difference between “in bed” and sleep onset [time of falling asleep]), sleep efficiency (TST/time spent in bed [“in bed” to “out of bed”]), and average awakening length (min; average duration of wakefulness per awakening). These sleep-related variables were categorized, according to their respective characteristics, into two groups: sleep profile (sleep onset latency, TMB, TST, and sleep efficiency) and fragmentation indices (WASO, number of awakenings, and average awakening length) [17]. For measurement of sleep quality, we chose the PSQI (Japanese version) because it is the most widely used general assessment of sleep quality in clinical and research settings [18]. The PSQI is used to calculate a global score and to derive categorical scores for its seven components: sleep quality, sleep latency, sleep duration, sleep efficiency, sleep disturbances, use of sleep medication, and daytime dysfunction [19]. PSQI scores ≥ 5 indicate poor sleep quality and high levels of sleep disturbances in athletes [17]. Furthermore, all participants were required to complete a sleep log, including their wake-up and bedtimes, using Google Forms™ (Google LLC, Mountain View, CA, USA). These data were collected in the same week as the training load measurement.

### 2.4. Hormone Analyses

Saliva samples were collected using the EIA kit (Salimetrics^®^, Carlsbad, CA, USA) on Monday afternoon (team day off) during the same week as the training load measurement. Samples were kept chilled, rapidly frozen within an hour, and subsequently stored until analysis. The cortisol (ug/dL) and testosterone (pg/mL) levels were analyzed, and the testosterone-to-cortisol ratio was calculated to assess the balance between anabolic and catabolic processes.

### 2.5. Statistical Analysis

All analyses were performed using IBM^®^ SPSS^®^ Statistics (version 24.0; IBM Corp., Armonk, NY, USA). All data were examined for normality using the Shapiro–Wilk test. If data were not normally distributed, nonparametric statistics were applied. The physical traits, training loads (internal and external), sleep-related variables, and hormone levels are presented as mean, standard deviation, range, and 95% confidence intervals. The effect size was calculated for all ANOVAs using partial eta-squared, with 0.01 considered a small effect size, 0.06 a medium effect size, and 0.15 a large effect size [20].

The reproducibility of measurements for internal and external training loads, sleep-related variables, and hormone levels was verified with the intraclass correlation coefficient and standard error of measurements [21]. Individual one-way repeated-measures ANOVAs were conducted for normally distributed data, and Friedman’s tests were used for non-normally distributed data. When a significant main effect of phase was observed, Scheffe’s test (for normally distributed data) or Bonferroni correction (for non-normally distributed data) was used to compare the four phases. Pearson correlation coefficient (for normally distributed data) or Spearman rank-sum test (for non-normally distributed data) was used to examine the associations between internal and external training loads, hormone levels, and sleep-related variables. 95% confidence intervals (CIs) for correlation coefficients were estimated using Fisher’s z transformation in Python^®^ 3. Multiple regression analyses were conducted using both stepwise and forced entry methods to examine factors affecting sleep parameters. Partial correlation coefficients (pccs) were calculated to assess the independent relationships between predictor variables and sleep parameters. The percentage of variance explained by the model for each variable was represented as ΔR^2^. Significance was set at 0.05. For the Bonferroni correction, this alpha level was adjusted to 0.0125.

## 3. Results

The Shapiro–Wilk test indicated that all variables, except testosterone, followed a normal distribution. We confirmed a significant main effect of time for session RPE (AM), ABELIs (AM and PM), number of awakenings, PSQI, and cortisol (*p* < 0.01, 0.01, 0.01, 0.05, 0.05, and 0.05, respectively) (Supplementary Table S1). Moderate-to-strong correlations were observed between session RPE (AM and PM) and sleep latency (r = 0.746, *p* < 0.01; r = 0.615, *p* < 0.05, respectively). Additionally, session RPE (AM) had a significantly positive relationship with TST (r = −0.667, *p* < 0.05) in October. Additionally, ABELIs (AM) had a significantly positive relationship with the number of awakenings (r = 0.630, *p* < 0.05), whereas cortisol levels had a negative relationship with TST (r = −0.642, *p* < 0.05). In November, testosterone levels were positively related with sleep latency (r = 0.606, *p* < 0.05), and cortisol levels had a strong positive correlation with TST (r = 0.836, *p* < 0.01). Furthermore, session RPE (PM) had a significant positive relationship with PSQI (r = 0.603, *p* < 0.05). The December results indicated a significant positive correlation between session RPE (AM) and sleep efficiency (r = 0.691, *p* < 0.05). WASO was strongly negatively correlated with session RPE (AM) (r = −0.698, *p* < 0.05), and the number of awakenings also had a negative relationship with session RPE (AM) (r = −0.785, *p* < 0.01). In January, ABELIs (AM) had a significantly positive relationship with PSQI (r = 0.607, *p* < 0.05), whereas testosterone levels were positively correlated with sleep latency (r = 0.603, *p* < 0.05). Moreover, ABELIs (PM) exhibited a strong negative relationship with sleep latency (r = −0.703, *p* < 0.05) (Supplementary Table S2). Multiple linear regression analyses showed factors significantly influencing sleep parameters. Sleep latency was significantly related to testosterone levels (pcc = 0.336, *p* = 0.026, ΔR^2^ = 0.113). Sleep efficiency was negatively correlated with ABELIs (AM) (pcc = −0.328, *p* = 0.029, ΔR^2^ = 0.108). TMB was significantly estimated by session RPE (AM) (pcc = −0.366, *p* = 0.015, ΔR^2^ = 0.134). TST was negatively related to ABELIs (AM) (pcc = −0.36, *p* = 0.016, ΔR^2^ = 0.13). WASO was significantly related to both ABELIs (AM) (pcc = 0.549, *p* < 0.001, ΔR^2^ = 0.266) and session RPE (AM) (pcc = −0.489, *p* = 0.002, ΔR^2^ = 0.194). The number of awakenings was also related to ABELIs (AM) (pcc = 0.434, *p* = 0.003, ΔR^2^ = 0.188), whereas average awakening length was significantly related to ABELIs (AM) (pcc = 0.39, *p* = 0.015, ΔR^2^ = 0.139) (Supplementary Table S3).

## 4. Discussion

This study was conducted primarily during the off-season and targeted male collegiate rowers to examine how internal and external training loads and hormonal markers influence sleep-related parameters. To investigate these relationships, multiple significant results were obtained through correlation analysis, ANOVA, and multiple regression analyses, although this discussion primarily focuses on the relationships among training load, hormonal fluctuations, and key sleep parameters, including both sleep profile (sleep onset latency, TMB, TST, and sleep efficiency) and fragmentation indices (WASO, number of awakenings, and average awakening length).

### 4.1. Sleep Profile

Correlation analysis showed that sleep latency was significantly associated with session RPE (AM, PM) in October and ABELIs (PM) in January. According to ANOVA, session RPE (AM) in October, November, and December; ABELIs (AM) in October and November; ABELIs (PM) in November; and PSQI in October were all significantly higher than in other months. In a cross-sectional study using the PSQI, Halson et al. [19] reported that, in elite-level athletes, higher sleep latency contributed to higher total scores, indicating poorer overall sleep quality. Meanwhile, Kölling et al. reported that weeks with increased training intensity had negative effects on sleep in rowers, with shorter sleep latency being associated with greater sleep satisfaction [22].

In line with those findings, this study also indicated that the increased training load, especially in October, may have negatively impacted sleep quality during the same month, which supports our hypothesis. In particular, nighttime training has been reported to enhance sympathetic nervous system activity, leading to a negative effect on sleep quality [23], which may require careful attention when planning training programs. Other factors related to latency, the results of correlation analyses in November and January, and multiple regression analyses, which showed a tendency for higher testosterone levels to be associated with longer sleep onset latency. This finding may be explained by the increase in testosterone levels, which promotes dopamine release [24] and has been directly associated with disruptions in sleep patterns [25], potentially altering sleep architecture and resulting in excessive daytime sleepiness [26]. Correlation and multiple regression analyses showed that TST was negatively associated with session RPE (AM) in October and ABELIs (AM).

Additionally, multiple regression analyses revealed a negative association between TMB and session RPE (AM). As described above, internal and external training load, as well as PSQI, reached higher values in October compared with other months. Hausswirth et al. reported that sleep quality and sleep quantity (including actual sleep time) significantly decreased during the overload period [27]. These phenomena have been observed in endurance athletes who experienced overreaching or overtraining, and insufficient recovery is considered a potential cause [27]. We obtained comparable results, indicating a decline in both sleep quality and quantity during the period of increased training load in October. Sargent et al. reported that training start time had a significant effect on TST, with earlier start times being associated with shorter TST [28]. In general, it is customary for rowers, including those in the present study, to start training early in the morning [19], and our participants also woke up between 4:30 and 5:30 AM, which is also likely to be a key factor contributing to their decreased TST.

Furthermore, TST was negatively correlated with cortisol levels in October, whereas it was positively correlated in November. Additionally, cortisol levels in January were significantly higher than in November according to ANOVA. Importantly, session RPE (PM) in November and ABELIs (AM) in January showed a significant positive correlation with PSQI. Partially in line with our findings, Perrier et al. reported that evening moderate-intensity aerobic exercise tended to shorten TST and increase cortisol secretion [29]. Taken together, our findings highlight that increased training load negatively affected sleep quality and quantity. Notably, the increase in cortisol levels observed in November was associated with an increase in TST, consistent with a previous study [29], suggesting a potential physiological stress response resulting from insufficient recovery. This phenomenon may indicate an elevated need for recovery due to accumulated physical or physiological stress. Regarding sleep efficiency, multiple regression analysis revealed a negative partial correlation with ABELIs (AM), whereas a significant positive correlation was observed with session RPE (AM) in December.

Furthermore, according to ANOVA, December ABELIs were significantly lower compared with October and November in the morning, and compared with November in the evening. A decline in sleep efficiency has been associated with overreaching, overtraining [5], and injuries [30] and is therefore considered undesirable. Thus, regular monitoring may be important for properly managing sleep efficiency throughout the season by appropriately balancing training load and recovery, given that overreaching and overtraining [5], which are often the result of inadequate recovery, can directly lead to poor performance [31]. Interestingly, despite the observed reduction in external load (ABELI) in December, a positive correlation between session RPE and sleep efficiency still emerged, partially aligning with our hypothesis. This issue will be further discussed below.

### 4.2. Fragmentation Indices

The current findings demonstrate that external training load (ABELI in AM) was positively associated with WASO, the number of awakenings, and the average awakening length, as shown by multiple regression analysis. Furthermore, ABELI (AM) was significantly higher in October and November than in December and January and was positively correlated with the number of awakenings in October. This result suggests that higher levels of external physical stress may contribute to more frequent and prolonged nocturnal awakenings.

These results are consistent with a study reporting that increased physical load can disturb sleep continuity (i.e., decrease immobility time), possibly because of the presence of mild muscle fatigue and increased soreness [27]. Frequent awakenings and extended periods of wakefulness during the night are recognized markers of diminished sleep quality, potentially impairing recovery in athletes [17]. Similar to the alterations observed in the sleep profile, fragmentation indices also exhibited deterioration in response to increased training load, with the most pronounced effects occurring in October, a month characterized by elevated physical demands. In contrast, as described above, AM and PM ABELIs were significantly lower in December versus October and November. Additionally, the number of awakenings was significantly lower in December versus both October and January. Further, session RPE (AM) in December was negatively correlated with both WASO and the number of awakenings, as confirmed by the multiple regression analysis for WASO.

This phenomenon appears somewhat paradoxical, especially as internal load showed a negative correlation with WASO and the number of awakenings despite the external load being reduced compared with other months. Kårström et al. [32] reported that the internal and external training loads of adolescent biathletes exhibited different trends over the same period, and the authors thus emphasized the importance of considering both internal and external load indicators when evaluating or designing training programs [32]. Overall, our results suggest that external load negatively influences sleep-related fragmentation indices, whereas internal load appeared to have a beneficial impact such as decreasing the number of awakenings. These findings are consistent with our hypothesis that internal and external loads differentially affect specific sleep indices. Moreover, correlation analyses conducted for each month revealed that the effects of internal and external loads on sleep-related variables differed depending on the month. Therefore, when assessing athletes’ recovery status, especially sleep, it is important to carefully consider the impacts of both internal and external training loads. Further, to address such sleep disturbances, consideration should be given to strategies such as sleep extension, scheduled napping, delaying the start time of early-morning training, and consistent enforcement of optimal sleep hygiene practices [28].

There are some potential limitations to this study. First, it included only 11 male collegiate rowers, resulting in a relatively small and homogeneous sample. Although the statistical power was sufficient, the generalizability of the findings to broader athletic populations remains limited. Second, all participants belonged to the same university rowing club, implying similar athletic performance levels and lifestyle patterns. Therefore, the results may not be applicable to athletes from other sports, female athletes, or individuals in other age groups. Third, the study was conducted exclusively during the off-season period, from October to January. As such, the findings may not be generalizable to in-season or competition phases, which could involve different training intensities and stress profiles. Finally, the correlation analyses were based on a small sample size for each month (*n* = 11), which may limit the statistical stability of the observed relationships. Therefore, these correlations should be interpreted with caution, as they do not indicate causality but rather reflect potential associations that warrant further investigation.

From a practical perspective, these findings may help coaches and sports scientists design training programs that better balance training load and recovery, particularly by monitoring modifiable sleep parameters such as TST and TMB. Future research should investigate these relationships in larger and more diverse athlete populations, including female athletes and other sports disciplines, to enhance generalizability. In addition, longitudinal or interventional studies are warranted to clarify causal mechanisms underlying the associations observed in this study. Despite the relatively small and homogeneous sample, a strength of this study lies in its comprehensive assessment of internal and external training loads, sleep parameters, and hormonal markers over multiple months during the off-season. This approach provides valuable insight into how these factors interact dynamically over time.

## 5. Conclusions

This study examined the relationships between internal and external training loads, hormonal fluctuations, and sleep-related parameters in collegiate male rowers during the off-season. Training load generally influenced various aspects of sleep, with the impact being most pronounced in October, a month characterized by elevated training load, which coincided with the most notable declines in both sleep quantity and quality. However, monthly analyses indicated that internal and external loads exerted different effects on sleep, highlighting the importance of regularly monitoring the interactions between these load types and sleep parameters over time. Among the various sleep-related indicators, quantitative measures such as TST and TMB emerged as modifiable factors, suggesting that these parameters may serve as practical targets for recovery-oriented interventions. Strategies such as delaying early-morning training sessions (i.e., sleep extension) or incorporating daytime naps could be effective in improving sleep quantity and, consequently, recovery and athletic performance. Additionally, hormonal fluctuations, particularly in cortisol and testosterone, were associated with variations in sleep latency and TST, suggesting a potential physiological mechanism linking training stress and the sleep architecture. The observed associations of both internal and external load indicators with sleep parameters suggest the utility of combining subjective and objective measures for non-invasive recovery monitoring. Given that internal and external loads appear to influence sleep in different ways, it is important to assess athletes’ recovery status with careful consideration of the distinct contributions of each type of load.

## Figures and Tables

**Table 1:** Physical traits of Japanese collegiate rowers (*n* = 11).

	Mean ± SD	Range	95% CI
Height (cm)	176.8 ± 3.6	172.0–182.0	174.4–179.2
Body mass (kg)	74.6 ± 3.8	68.3–82.0	72.1–77.1
Age (year)	21.2 ± 0.8	20.0–22.0	20.7–21.7
Rowing years	3.9 ± 1.1	3.0–6.0	3.2–4.6

Abbreviations: CI, confidence interval; SD, standard deviation.

## Data Availability

Data are available on request.

## Supplementary Materials

The following supporting information can be downloaded at https://www.mdpi.com/article/10.3390/sports13110407/s1: Table S1. Training volume by type and season. Descriptive statistics (mean ± SD, range, and 95% CI) of training volume variables (e.g., session-RPE) across months (October–January) with main effects and post hoc comparisons. Table S2. Correlation coefficients (r) with 95% confidence intervals (CI) between sleep parameters, training load, and hormonal levels across four months. Correlation analyses showing relationships among sleep variables, training-load indices, and hormonal measures. Table S3. Factors affecting parameters related to sleep. Multiple regression analyses using stepwise and forced entry methods to examine factors associated with sleep parameters (pcc and ΔR^2^ shown).

## Abbreviations

The following abbreviations are used in this manuscript:

RPE	Rating of perceived exertion
ABELIs	Accelerometry-based external load indicators
PSQI	Pittsburgh Sleep Quality Index
TMB	Total minutes in bed
TST	Total sleep time
WASO	Wake after sleep onset
pccs	Partial correlation coefficients
CI	Confidence interval
SD	Standard deviation
